# Are recent attempts to quit smoking associated with reduced drinking in England? A cross-sectional population survey

**DOI:** 10.1186/s12889-016-3223-6

**Published:** 2016-07-22

**Authors:** Jamie Brown, Robert West, Emma Beard, Alan Brennan, Colin Drummond, Duncan Gillespie, Matthew Hickman, John Holmes, Eileen Kaner, Susan Michie

**Affiliations:** Department of Clinical, Educational and Health Psychology, University College London, London, UK; Cancer Research UK Health Behaviour Research Centre, University College London, London, UK; UK Centre for Tobacco and Alcohol Studies (UKCTAS), Nottingham, UK; National Centre for Smoking Cessation and Training, London, UK; Sheffield Alcohol Research Group, ScHARR, The University of Sheffield, Sheffield, UK; Institute of Psychiatry, Psychology and Neuroscience, King’s College London, London, UK; School of Social and Community Medicine, University of Bristol, Bristol, UK; Institute of Health & Society, Newcastle University, Newcastle, UK

**Keywords:** Alcohol drinking, Attempts, Cutting down, Smoking, Smoking cessation

## Abstract

**Background:**

Alcohol consumption during attempts at smoking cessation can provoke relapse and so smokers are often advised to restrict their alcohol consumption during this time. This study assessed at a population-level whether smokers having recently initiated an attempt to stop smoking are more likely than other smokers to report i) lower alcohol consumption and ii) trying to reduce their alcohol consumption.

**Method:**

Cross-sectional household surveys of 6287 last-year smokers who also completed the Alcohol Use Disorders Identification Test consumption questionnaire (AUDIT-C). Respondents who reported attempting to quit smoking in the last week were compared with those who did not. Those with AUDIT-C≥5 were also asked if they were currently trying to reduce the amount of alcohol they consume.

**Results:**

After adjustment for socio-demographic characteristics and current smoking status, smokers who reported a quit attempt within the last week had lower AUDIT-C scores compared with those who did not report an attempt in the last week (β_adj_ = −0.56, 95 % CI = −1.08 to −0.04) and were less likely to be classified as higher risk (AUDIT-C≥5: OR_adj_ = 0.57, 95 % CI = 0.38 to 0.85). The lower AUDIT-C scores appeared to be a result of lower scores on the frequency of ‘binge’ drinking item (β_adj_ = −0.25, 95 % CI = −0.43 to −0.07), with those who reported a quit attempt within the last week compared with those who did not being less likely to binge drink at least weekly (OR_adj_ = 0.54, 95 % CI = 0.29 to 0.999) and more likely to not binge drink at all (OR_adj_ = 1.70, 95 % CI = 1.16 to 2.49). Among smokers with higher risk consumption (AUDIT-C≥5), those who reported an attempt to stop smoking within the last week compared with those who did not were more likely to report trying to reduce their alcohol consumption (OR_adj_ = 2.98, 95 % CI = 1.48 to 6.01).

**Conclusion:**

Smokers who report starting a quit attempt in the last week also report lower alcohol consumption, including less frequent binge drinking, and appear more likely to report currently attempting to reduce their alcohol consumption compared with smokers who do not report a quit attempt in the last week.

## Background

Smoking and excessive alcohol consumption are two of the most serious public health problems [1–4] and have a close and complex relationship [5]. There is co-morbidity such that people with an alcohol disorder are substantially more likely to smoke cigarettes [6–10]. Cross-sectional epidemiological data indicate that lifetime quit rates for smokers are lower [5–7] and nicotine dependence higher in those with an alcohol use disorder [5], while longitudinal data indicate that smokers with an alcohol use disorder are less likely to attempt and succeed in stopping [11, 12] and among former smokers, those with an alcohol use disorder appear more likely to relapse [11]. Alcohol consumption during attempts at smoking cessation is associated with greater risk of lapse and relapse [13–17] even after adjusting for socio-demographic characteristics and tobacco dependence [13]. Alcohol-related lapses also appear qualitatively distinct from lapses not involving alcohol [13]. There are variety of possible mechanisms through which alcohol consumption could increase the risk of lapse during a smoking cessation attempt (for example, reduction in self-control, increased salience of smoking cues or increased likelihood of exposure to other smoking cues; [17]), and as a consequence smokers are advised to restrict their alcohol consumption during an attempt to stop [13, 18, 19].

This position is supported by the wider multiple health behaviour change literature. The field of multiple behaviour change is motivated by findings that many health behaviours tend to cluster and often result in synergistic effects on mortality and morbidity [20, 21]. In theory this presents a cost-effective opportunity to target particularly at-risk groups and intervene on several public health problems simultaneously. In practice, multiple behaviour change has proved a considerable challenge [22, 23]. While generalisable results are scarce, a recent theoretically-guided meta-analysis of behaviour change interventions indicated that there may be a curvilinear relation between the number of behavioural recommendations and outcome, with a moderate (2–3) number producing the greatest effect [24].

It is unclear at a population-level whether smokers follow advice to restrict alcohol during a quit attempt. One indicator would be the association between a recent attempt to stop smoking and alcohol consumption. It is not possible with cross-sectional epidemiological data to rule out the possibility that any association could be explained by people with lower alcohol consumption being more likely to attempt to quit smoking. However, the association is important whichever the direction of causality; and this alternative may suggest the need for smokers with higher alcohol consumption to be encouraged to quit smoking. To help tease apart the issue, it is also instructive to assess whether among smokers with higher-risk alcohol consumption there is an association between a recent attempt to quit smoking and a current attempt to cut down alcohol consumption.

This study sought to address the following research questions: What is the association among smokers in England between a recent attempt to quit smoking and alcohol consumption? What is the association among smokers with higher risk alcohol consumption in England between a recent attempt to stop smoking and a current attempt to cut down on their drinking?

## Methods

### Study design

Data were collected using repeated cross-sectional household surveys of representative samples of the population of adults in England conducted in consecutive monthly waves between March 2014 and September 2015. The surveys are part of the ongoing Smoking Toolkit Study (STS) and Alcohol Toolkit Study (ATS) which are designed to provide tracking information about smoking, alcohol consumption and related behaviours in England [25, 26]. Each month a new sample of approximately 1700 adults aged 16 and over complete a face-to-face computer-assisted survey. The sampling is a hybrid between random probability and simple quota and the method has been shown to result in a sample that is nationally representative in its socio-demographic composition [25].

### Study population

We used data from respondents aged 16 and over in the period from March 2014 to September 2015 who reported smoking tobacco in the last year by endorsing one of the first four options on the following question: ‘Which of the following best applies to you? Please note cigarettes refer to tobacco and not electronic cigarettes.’: (i) ‘I smoke cigarettes (including hand-rolled) everyday’; (ii) ‘I smoke cigarettes (including hand-rolled) but not every day’; (iii) ‘I do not smoke cigarettes at all but I do smoke tobacco of some kind (e.g., pipe or cigar)’; (iv) I have stopped smoking completely in the last year’; (v) I stopped smoking completely more than a year ago; (vi) I have never been a smoker (i.e., smoked for a year or more).

### Measures

Alcohol consumption was assessed by the first three consumption questions on the widely validated Alcohol Use Disorders Identification Test (AUDIT-C; [27]). The AUDIT-C is the short-form of the ten-item AUDIT questionnaire and provides a score ranging between 0 and 12 (with 0 indicating non-drinkers). For the current study, higher risk consumption was indicated by a score ≥ 5 [28]. The three component items of the AUDIT-C consist of ‘drinking frequency’ (0–4), ‘typical quantity per occasion’ (0–4), and ‘high intensity or ‘binge’ drinking frequency’ (0–4). From the binge drinking item, categorical measures were derived of those who did not binge drink compared with those who did, and those who did binge drink at least weekly compared with those who did not. Smokers whose consumption of alcohol was classified as higher risk, were also asked whether they were currently attempting to restrict their alcohol consumption.

All smokers were asked whether they had made a serious attempt to stop smoking and those who had made an attempt were asked how recently. Smokers were classified into those who attempted to stop in the last week and those who had not. Respondents were also asked questions that assessed: age; sex; an occupationally-based classification of socio-economic status called ‘social grade’ (dichotomised to ABC1 = higher and intermediate professional/managerial and supervisory, clerical, junior managerial/administrative/professional or C2DE = skilled, semi-skilled, unskilled manual and lowest grade workers or unemployed); government office region in England (dichotomised to North = North East, North West, and Yorkshire and the Humber, East Midlands, West Midlands, or South = East of England, London, South East, and South West, classified according to an established North-South divide [29]); receipt of a voluntary educational qualification (obtained after compulsory education ceases at 16 years old); ethnicity; and disability.

### Analysis

To examine associations between indices of alcohol consumption and a recent attempt to quit smoking, we first constructed unadjusted regression models in which we regressed i) indices of alcohol consumption onto a recent attempt to quit smoking (reference: no attempt to quit within the last week). We used linear regression models for the continuous dependent variables (scores on the AUDIT-C, and scores on the component items ‘drinking frequency’, ‘typical quantity per occasion’, and ‘binge frequency’) and logistic regression for the binary dependent variables (proportion classified as high risk AUDIT-C, binge drinking at least weekly, and no binge drinking). To examine the independent association after adjustment with each dependent variable, we then constructed multivariable regression models including also the socio-demographic and smoking variables listed in Table 1 (age, sex, social grade, region, receipt of a voluntary educational qualification, ethnicity, disability and current smoking status) and the survey wave. In a final subgroup analysis, to examine associations between a recent attempt to stop smoking and a current attempt to cut down drinking among smokers with higher risk alcohol consumption, we first constructed an unadjusted logistic regression model in which we regressed a current attempt to cut down drinking onto a recent attempt to quit smoking (reference: no attempt to quit within the last week). To examine the independent association after adjustment, we constructed a multivariable logistic regression model including also the socio-demographic variables listed in Table 1 and the survey wave.

**Table 1:** Socio-demographic characteristics of last-year smokers by recent quitting

	No quit attempt within last week (*N* = 6143)	Quit attempt ≤ 1 week (*N* = 144)	t/*χ* ^2^-value (d.f.), *p*-value	Total (*N* = 6287)
Mean (SD) age	42.6 (17.2)	41.5 (16.0)	0.75 (6285), 0.46	42.5 (17.2)
% (N) Women	46.3 (2842)	47.9 (69)	0.16 (1), 0.69	46.3 (2911)
% (N) Lower social grade (C2DE)	63.0 (3872)	65.3 (94)	0.30 (1), 0.58	63.1 (3966)
% (N) North region	58.1 (3572)	56.3 (81)	0.21 (1), 0.65	58.1 (3653)
% (N) No voluntary educational qualifications	46.3 (2842)	47.2 (68)	0.05 (1), 0.82	46.3 (2910)
% (N) White	89.2 (5479)	85.4 (123)	2.06 (1), 0.15	89.1 (5602)
% (N) Disability	15.1 (928)	16.7 (24)	0.27 (1), 0.61	15.1 (952)
% Currently not smoking	5.6 (346)	24.3 (35)	86.2 (1), <0.001	6.1 (381)

## Results

A total of 31,878 adults in England were surveyed between March 2014 and September 2015, of whom 6652 (20.9 %) reported smoking in the last year and 6287 (94.5 %) of these smokers also provided complete data on all variables. The overall socio-demographic characteristics of the sample (see Table 1) were broadly representative of smokers: the sample was younger and contained a lower proportion of women and greater proportion of people with lower social grade and no post-16 qualifications than would be expected in a general population sample [25]. A total of 144 of these smokers reported that they had attempted to quit smoking in the last week. There was no evidence of association between recent quitting and socio-demographic characteristics but, as expected, those last-year smokers who had attempted to quit in the last week were much more likely to report currently not smoking than those who had not attempted to quit within the last week (see Table 1).

**Table 2:** Associations between a recent attempt to quit smoking and i) indices of alcohol consumption and ii) attempts to cut down on alcohol consumption among the subgroup of high-risk drinkers

Mean (95 % CI)	No quit attempt within last week (*N* = 6143; reference)	Quit attempt ≤ 1 week (*N* = 144)	B (95 % CI), *p*-value	B_adj_ (95 % CI), *p*-value
AUDIT-C (0–12)	3.46 (3.38 to 3.54)	2.80 (2.31 to 3.29)	−0.66 (−1.21 to −0.11), 0.02	−0.56 (−1.08 to −0.04), 0.03
AUDIT 1: Drinking frequency (0–4)	1.63 (1.60 to 1.67)	1.42 (1.19 to 1.65)	−0.21 (−0.45 to 0.02), 0.08	−0.14 (−0.36 to 0.09), 0.24
AUDIT 2: Typical quantity (0–4)	0.98 (0.95 to 1.01)	0.80 (0.60 to 0.99)	−0.18 (−0.40 to 0.04), 0.10	−0.17 (−0.38 to 0.03), 0.10
AUDIT 3: Binge frequency (0–4)	0.84 (0.82 to 0.87)	0.58 (0.42 to 0.74)	−0.27 (−0.46 to −0.08), 0.01	−0.25 (−0.43 to −0.07), 0.01
% (N)			OR (95 % CI), *p*-value	OR_adj_ (95 % CI), *p*-value
High-risk AUDIT-C (≥5)	36.3 (2230)	25.0 (36)	0.58 (0.40 to 0.86), 0.01	0.57 (0.38 to 0.85), 0.01
At least weekly binge drinking	14.8 (908)	8.3 (12)	0.52 (0.29 to 0.95), 0.03	0.54 (0.29 to 0.999), 0.05
No binge drinking	57.6 (3541)	68.1 (98)	1.57 (1.10 to 2.23), 0.01	1.70 (1.16 to 2.49), 0.01
% (N) Subgroup analysis: AUDIT-C≥5	*N* = 2230	*N* = 36	OR (95 % CI), *p*-value	OR_adj_ (95 % CI), *p*-value
Attempts to cut down drinking	18.3 (409)	44.4 (16)	3.56 (1.83 to 6.93), <0.001	2.98 (1.48 to 6.01), <0.01

The adjusted models presented in the final column include all variables in Table 1 and wave of survey. The pattern of adjusted results was unchanged in sensitivity analyses in which the adjustment for social grade was entered into the models as a 5-level categorical variable or age was entered as a 6-level categorical variable instead of the dichotomised and continuous versions of the variables reported in Table 1 respectively

In an unadjusted model, smokers who had attempted to quit within the last week reported lower AUDIT-C scores than those who had not: scores were 0.66 points (95 % CI = 1.21 to 0.11) lower on average among those who had attempted to quit in the last week (see Table 2). This result was largely unchanged after adjustment for age, sex, social grade, region, voluntary educational qualification, ethnicity, disability, current smoking status and survey wave: the adjusted AUDIT-C score was 0.56 points (95 % CI = −1.08 to −0.04) lower among those who had attempted to quit in the last week (see Table 2). In both unadjusted and adjusted analyses, those smokers who had attempted to quit in the last week were also less likely to be classified as higher risk drinkers (AUDIT-C≥5, see Table 2).

The lower AUDIT-C scores among those who attempted to quit smoking in the last week appeared to be a result of lower scores on the component item [question 3] frequency of binge drinking (see Table 2), with those who begun a quit attempt in the last week being less likely to binge drink at least weekly and more likely to not binge drink at all (see Table 2). There was, however, no evidence of association between a recent attempt to quit smoking and the drinking frequency or typical quantity per occasion AUDIT-C items (see Table 2). In all these analyses, the results were broadly unchanged after adjustment (see Table 2).

The final subgroup analysis included only those smokers whose alcohol consumption was classified as higher risk (*n* = 2266). Compared with smokers who had not, those who had attempted to stop smoking within the last week were more likely to report also currently trying to restrict their alcohol consumption in both unadjusted and adjusted analyses (see Table 2).

## Discussion

This study found that smokers who reported attempting to stop within the last week had lower levels of alcohol consumption, especially bingeing, and were less likely to be classified as having higher risk alcohol consumption (AUDIT-C ≥5) compared with those who did not report an attempt to quit smoking in the last week. Among those with higher risk alcohol consumption, smokers who reported attempting to stop smoking within the last week compared with those who reported no attempt were more likely to report also currently trying to restrict their alcohol consumption.

This study adds to the literature on the close relationship between smoking and alcohol consumption [5]. One component of the relationship is that alcohol consumption is associated with lapse and relapse to smoking [13–17], which has resulted in smokers being widely advised to restrict their consumption during quit attempts [13, 18, 19]. In the current study, the association between a recent attempt to quit smoking and reduced alcohol consumption indicates that smokers in England may be following this best-practice advice to restrict their alcohol consumption during a smoking cessation attempt. It is not possible with cross-sectional epidemiological data to rule out reverse causation i.e., the possibility that the association between quitting and consumption may actually be driven by people with lower alcohol consumption being more likely to attempt to quit smoking. If this were the explanation, the association would remain important because it would suggest the need for smokers with higher alcohol consumption to be targeted for further encouragement to attempt to quit smoking. However, another finding in this study indicates that the association is unlikely to be driven exclusively by lighter drinkers being more likely to attempt quit smoking: among smokers who were also heavier drinkers, those who had made an attempt to quit smoking within the last week compared with those who had not were also more likely to report a current attempt to restrict their alcohol consumption. The present study cannot determine whether attempts to quit smoking tend to precede attempts to restrict alcohol consumption, or vice versa.

These findings have possible implications for policy evaluation and development: there appears to be a need for greater attention to possible crossover effects when evaluating the cost-effectiveness of alcohol and tobacco interventions and more reason for a coordinated strategy on alcohol and tobacco control. Policy on brief intervention by health professionals is one example. Brief intervention for smoking and alcohol are both effective and cost-effective interventions [30–34]. Delivery of smoking brief intervention is much more common in England than is alcohol [35], and there is a need to increase the rate of screening and brief intervention on alcohol [36, 37]. The current study suggests that smokers may be more likely to reduce their alcohol consumption when attempting to stop smoking than when they are not. While these findings cannot speak to the effectiveness of brief interventions, they do suggest that a smoking brief intervention may be a good opportunity to intervene also on alcohol: smokers may be likely to plan to reduce their alcohol consumption regardless and may therefore be particularly receptive to intervention on alcohol. However, this is an empirical question for which there is currently sparse experimental evidence [38–40], and until such evidence is forthcoming, other strategies to increase alcohol brief intervention may warrant greater resource [41–45]. In the meantime, the current findings could be simply disseminated to health professionals to reassure them that many smokers may be planning to cut down on their drinking anyway and their intervention on alcohol may be therefore unlikely to compromise the patient relationship: the GP-patient relationship is a regularly cited barrier to a greater rate of brief intervention, albeit one of several including inadequate training, and lack of time or financial incentives [46–52].

There are three important limitations of this study. The limitation on interpretation of cross-sectional design in relation to direction of causation has been discussed. A second limitation is that as an observational study it is possible that unmeasured confounding could have influenced the results. For example, it is possible that the diagnosis of a health problem led to attempts to cut down on both drinking and smoking (i.e., cross-behavioural sick-quitter effects). Our adjustment for a self-reported disability may not have sufficiently accounted for this possibility. Another limitation is that the study relied on self-reported data with the risk of socially desirable responding. However, in population surveys the social pressure and related misreporting of smoking is low and it is generally considered acceptable to rely on self-reported data [53], while we used an abbreviated version of a high quality tool that has been widely validated to assess alcohol use disorder (AUDIT-C; [27, 54]). The full version of the AUDIT questionnaire is more widely validated but includes questions across a longer reference period that would have rendered the scale less sensitive to recent changes, while AUDIT-C has good validity, excellent reliability and responsiveness to change [54].

## Conclusions

In conclusion, smokers who report a recent attempt to stop are more likely to report lower-risk alcohol consumption, including less frequent binge drinking, after adjusting for socio-demographic characteristics. Among smokers with higher-risk alcohol consumption, those who report a last week attempt to stop are more likely to report also a current attempt to cut down on their drinking.
